# Infection of rhesus macaques with a pool of simian immunodeficiency virus with the envelope genes from acute HIV-1 infections

**DOI:** 10.1186/s12981-016-0125-8

**Published:** 2016-11-25

**Authors:** Kendall C. Krebs, Meijuan Tian, Mohammed Asmal, Binhua Ling, Kenneth Nelson, Kenneth Henry, Richard Gibson, Yuejin Li, Weining Han, Robin J. Shattock, Ronald S. Veazey, Norman Letvin, Eric J. Arts, Yong Gao

**Affiliations:** 1 Division of Infectious Diseases, School of Medicine, Case Western Reserve University, 10900 Euclid Ave, Cleveland, OH 44106 USA; 2 Division of Viral Pathogenesis, Beth Israel Deaconess Medical Center, Harvard Medical School, Boston, MA 02115 USA; 3 Tulane National Primate Research Center, Tulane University School of Medicine, 18703 Three Rivers Road, Covington, LA 70433 USA; 4 Division of Medicine, Department of Infectious Diseases, Imperial College London, Norfolk Place, London, W2 1PG UK; 5 Department of Molecular Biology and Microbiology, School of Medicine, Case Western Reserve University, 10900 Euclid Ave, Cleveland, OH 44106 USA; 6 Department of Microbiology and Immunology, Schulich School of Medicine & Dentistry, University of Western Ontario, 1151 Richmond St., London, ON N6A 5C1 Canada

**Keywords:** Simian–human immunodeficiency chimeric virus, Transmission, Replicative fitness, Glycosylation

## Abstract

**Background:**

New simian–human immunodeficiency chimeric viruses with an HIV-1 env (SHIVenv) are critical for studies on HIV pathogenesis, vaccine development, and microbicide testing. Macaques are typically exposed to single CCR5-using SHIVenv which in most instances does not reflect the conditions during acute/early HIV infection (AHI) in humans. Instead of individual and serial testing new SHIV constructs, a pool of SHIVenv_B derived from 16 acute HIV-1 infections were constructed using a novel yeast-based SHIV cloning approach and then used to infect macaques.

**Results:**

Even though none of the 16 SHIVenvs contained the recently reported mutations in *env* genes that could significantly enhance their binding affinity to RhCD4, one SHIVenv (i.e. SHIVenv_B3-PRB926) established infection in macaques exposed to this pool. AHI SHIVenv_B viruses as well as their HIVenv_B counterparts were analyzed for viral protein content, function, and fitness to identify possible difference between SHIVenv_B3-PRB926 and the other 15 SHIVenvs in the pool. All of the constructs produced SHIV or HIV chimeric with wild type levels of capsid (p27 and p24) content, reverse transcriptase (RT) activity, and expressed envelope glycoproteins that could bind to cell receptors CD4/CCR5 and mediate virus entry. HIV-1env_B chimeric viruses were propagated in susceptible cell lines but the 16 SHIVenv_B variants showed only limited replication in macaque peripheral blood mononuclear cells (PBMCs) and 174×CEM.CCR5 cell line. AHI chimeric viruses including HIVenv_B3 showed only minor variations in cell entry efficiency and kinetics as well as replicative fitness in human PBMCs. Reduced number of N-link glycosylation sites and slightly greater CCR5 affinity/avidity was the only distinguishing feature of env_B3 versus other AHI env’s in the pool, a feature also observed in the HIV establishing new infections in humans.

**Conclusion:**

Despite the inability to propagate in primary cells and cell lines, a pool of 16 SHIVenv viruses could establish infection but only one virus, SHIVenv_B3 was isolated in the macaque and then shown to repeatedly infected macaques. This SHIVenv_B3 virus did not show any distinct phenotypic property from the other 15 SHIVenv viruses but did have the fewest N-linked glycosylation sites.

**Electronic supplementary material:**

The online version of this article (doi:10.1186/s12981-016-0125-8) contains supplementary material, which is available to authorized users.

## Background

A major hindrance in drug, vaccine, and microbicide development for HIV/AIDS is limited utility of existing animal models. Human immunodeficiency virus type 1 (HIV-1) productively infects only humans and chimpanzees. While chimpanzees can be productively infected, they are endangered, expensive, do not typically develop AIDS after HIV-1 infection, and their use in research engenders ethical concerns [1]. This narrow host range of HIV-1 has compelled researchers to use macaque monkeys exposed or infected with SHIVenv, a chimera containing HIV-1 env coding regions within a simian immunodeficiency virus (SIV) backbone derived from rhesus macaques (*Macaca mulatta*) infections. The genomic organization of SIVmac, HIV-1, and SHIV constructs are similar but encode for virus with significant functional variations. These differences are largely based on the accessory proteins, which appear to modulate viral replication in a host species-dependent manner impacting virus persistence, spread, and pathogenesis [2–5]. A recent study showed that the HIV harboring the SIVmac vif gene could establish infection and was pathogenic in pigtailed macaques (*Macaca nemestrina*) depleted for CD8+ T cells [6].

SIV strains containing HIV-1 *env* genes (SHIVenv) have been successfully employed to infect macaques through intravenous and mucosal routes. Currently, most SHIVenv’s are clonal and even following propagation, do not contain a diverse representative of the HIV-1 population transmitted from donor to establish infection in a recipient with a single HIV-1 clone. Lack of diverse SHIVenv populations as innocula for macaque infection studies represents a resource gap for the rational development of HIV-1 vaccines and testing of microbicides. It is also critical to establish new *env*-based SHIVs for studies on pathogenesis and immune responses. However, the prospect of developing new infectious SHIVenv viruses is daunting considering the time consuming cloning procedures, the need for high titer virus propagation in extraneous cell lines, and the cost of testing infectivity in macaque infectivity using a reiterative SHIV strain-by-strain approach.

New HIV-1 infections (60–90%) originate from single HIV-1 variant or a limited number of transmitted/founder HIV-1 variants despite exposure to hundreds or thousands of HIV-1 clones from the donor partner [7, 8]. This genetic bottleneck is less pronounced in individuals engaged in high-risk behaviors (anal-receptive intercourse or intravenous drug use) and in patients with ongoing sexually transmitted infections [9]. Notably, acute infection with a “heterogeneous” infecting HIV-1 population has been linked to more rapid disease progression [10]. As indicated above, most macaque models for primary HIV-1 infection involve exposure with only a single or highly homogeneous SHIVenv virus and do not reflect exposure to the highly heterogenous HIV-1 from donor to recipient. The HIV-1 clone(s) establishing primary infection in humans may have unique phenotypic properties from the inoculating HIV-1 population making it more apt for transmission. For example, transmitted virus preferentially employs CCR5 as co-receptor (R5 tropic) even though CXCR4 using HIV-1 may be present in the inoculating virus population. In the case of external pressure, use of preventative vaccines and microbicides should block new HIV-1 transmission. Prior to human trials, macaque models remain crucial for studies on HIV vaccine and microbicide testing [11, 12]. However, few CCR5-using SHIVenv strains (e.g. SHIV_SF162_, SHIV_CHN19_, SHIV_1157ipd3N4_) can maintain stable and prolonged infections [13–15]. A recent study used a pool of SHIVenv viruses to infect macaques depleted of CD8+ T cells resulting identification of two pathogenic SHIVenv strains [16]. Other studies suggested that the mutations in Env region could enhance macaque CD4-mediated entry and viral replication [17, 18]. However, how these mutations impact SHIV pathogenesis in macaques has not been fully explored.

There were two main objectives for this study and both required the initial construction of SHIVs containing the transmitted subtype B *env* genes, derived from the AHIs of CHAVI001 and other CHAVI clinical trials. The first objective was to identify R5 SHIVenv viruses with high transmission efficiency based on exposure of macaques to the heterogenous SHIVenv pool. As described herein, a single SHIVenv clone established macaque infection which prompted comprehensive genotypic and phenotypic analyses of why this SHIVenv was transmitted versus the other 15 in the pool. These analyses of transmission fitness required a battery of assays to measure proper virus assembly, replicative fitness, and the efficiency/kinetics of host cell entry, as well as transmission related modification (e.g. glycosylation) in these envelope proteins. The second objective in the companion article [19] was to establish a pathogenic R5 SHIV from the SHIVenv with the highest transmission efficiency. It is important to stress that transmission efficiency and pathogenicity is likely related to different virus attributes. In past, the serial strain-by-strain cloning then testing in macaques has failed to identify a pathogenic R5 SHIVenv that provides a macaque model for prolonged HIV-1 infection in humans. Thus, we have serially passaged the highly transmissible SHIVenv to develop a new pathogenic R5 SHIVenv.

## Methods

### Macaque use

Adult rhesus macaques (*Macaca mulatta*) were housed at the New England Primate Research Center and Harvard Medical School, a primate animal facility that is accredited by the Association for the Assessment and Accreditation of Laboratory Animal Care International. Research was conducted in compliance with the Animal Welfare Act and other US federal statutes and regulations relating to animals and experiments involving animals, and adhered to principles stated in the Guide for the Care and Use of Laboratory Animals, National Research Council, 1996. All steps were taken to ameliorate the welfare and to avoid the suffering of the animals in accordance with the “Weatherall report for the use of non-human primates” recommendations. Animals were housed either socially or in adjoining individual primate cages allowing social interactions, under controlled conditions of humidity, temperature and light (12-h light/12-h dark cycles). Food and water were available ad libitum. Animals were fed commercial monkey chow and treats by trained personnel. Environmental enrichment consisted of commercial toys. Blood draws were conducted under sedation by trained personnel under the supervision of veterinarian.

### Yeast strain and growth conditions

*Saccharomyces cerevisiae* Hanson (MYA-906), MAT alpha ade6 can1 his3 leu2 trp1 URA3, was obtained from the American Type Culture Collection (ATCC). Yeast was grown at 30 °C in appropriate media depending on the cloning step [yeast extract peptone dextrose (YEPD), complete (C) minimal media -LEU-URA3, C-LEU, or C-LEU/5-fluoro-1,2,3,6-tetrahydro-2,6-dioxo-4-pyrimidine carboxylic acid (5-FOA)]. Transformations were performed using the lithium acetate (LiAc) method. Briefly, the vector DNA and PCR product (~3 μg) were added to competent cells at a 1:3 ratio along with 50 µg of single stranded salmon sperm carrier DNA (BD Biosciences/Clontech, Palo Alto, CA, USA) and sterile polyethylene glycol (50%)/TE (10 mM Tris–Cl, 1 mM EDTA)/LiAc (100 mM). Following agitation for 30 min at 30 °C, yeast was heat shocked at 42 °C for 15 min and plated on C-leu agar plates containing the appropriate selection.

### Cell culture

PBMCs were isolated from HIV-1 seronegative donors and macaques via Ficoll-Paque density centrifugation and cultured in a RPMI-1640 medium with 10% fetal bovine serum (FBS). PBMCs were obtained from donors with written informed consent in the Cleveland area under the UH IRB approval AIDS125 (protocol #01-98-55). All informed consent documents were stored in locked, secure cabinet and only the person authorized by the protocol to obtain informed consent from the participant has access to this locked cabinet. The protocol is renewed yearly but the year of analyses and collection for this study had the expiry date of 06/20/2014. Children were not used in this study. The U87.CD4.CXCR4 cell line was obtained from the AIDS Research and Reference Reagent Program and grown in Dulbecco’s modified Eagle’s medium (DMEM, Cellgro) supplemented with 15% FBS, penicillin and streptomycin, puromycin (1 μg/ml) and G418 sulfate (1 mg/ml). 293T cells were obtained from the American Type Culture Collection and grown in DMEM supplemented with 10% FBS and penicillin and streptomycin. 174×CEM.CCR5 cells (kindly provided by Dr. Nathaniel Landau at SOM, NYU) were grown in RPMI-1640 containing 10% FBS, penicillin and streptomycin, 1%HEPES, Puromycin (0.5 μg/ml), G418 (0.3 mg/ml), and Hygromycin B (200 μg/ml). All cells were grown at 37 °C in 5% CO_2_.

### Molecular clones

Twenty acute subtype B envelopes (*env*, provided by Drs. Keele and Hahn, University of Alabama) were cloned from the CHAVI acute infection studies [7]. Three chronic subtype B *env*s (i.e. I10, K44, and Q0—isolated from patients infected at one to 2 years post infection) were cloned from anonymous patient samples under IRB approval #07-02-32 at Case Western Reserve University/University Hospitals of Cleveland IRB. HIV-1 *env*s were cloned using the protocol described in [20]. Approximately 3 μg of the 2 Kbp *env* DNA (HXB2 numbering 6108-8082) from acute infection clones (or from chronic infections) were transfected into yeast with linearized plasmids, pREC_nfl_SHIV_KB9__Δenv/URA3, or pREC_nfl_HIV_Δenv/URA3. Yeast colonies were selected on C-leu plates supplemented with FOA but lacking leucine. Approximately 95% of FOA-resistant yeast colonies harbored pREC_nfl_SHIV_KB9_ or pREC_nfl_HIV with the patient *env*. A crude yeast lysate was then used to transform bacteria and to amplify these ampicillin-resistant DNA plasmids for purification as described [20]. It is important to note that, for the chronic patient samples, the *env* PCR product contained the amplified HIV-1 population in that patient sample and as such, >100 yeast colonies were removed from C-leu/FOA plates for bulk plasmid purification and for eventual reconstitution of HIV-1 *env* quasispecies. In contrast, only a single acute *env* clone was subcloned into pREC_nfl_SHIV_KB9__ Δenv/URA3 or pREC_nfl_HIV_ Δenv/URA3; this represents the limited genetic diversity in the acute sample. It is also important to stress that SHIV_KB9_ is a SIVmac239 virus containing only a portion of HIV-1 env gene, i.e. from the 1st exon of Tat to the end on the 2nd exon of Tat (HXB2 numbering nt5823-nt8676). The URA3 gene replaces in the pREC_nfl_HIV_Δenv/URA3 only replaces a region still flanked by KB9 sequence (HXB2 numbering nt5996-nt8638). In contrast, the SHIVenv vectors and virus contain the AHI subtype B env coding sequence within the KB9 tat1/gp120/tat2 coding sequence (HXB2 numbering 6108-8082).

### Production of SHIVenv, and HIVenv chimeric viruses

The vectors, pREC_nfl_SHIVenv_X (i.e. with patient X *env*) were then co-transfected into 293T cells with the complementing vector, pREC_cplt_R/U5/gag/pol [21], using Effectene Transfection Reagent (QIAGEN). As previously described [20], these co-transfected 293T cells produced virus particles harboring either two complement RNA genomes (SHIV_cplt_R/U5/gag/Δpol RNA), two near full length SHIV genomes (nfl_SHIVenv_X RNA), or both. Only the latter can propagate in susceptible cells or animals. Supernatant containing these three forms of viruses was harvested from the transfected 293T cells at 48 h, monitored for RT activity, and used for virus propagations in various susceptible cell lines (U87.CD.CCR5, 174×CEM.CCR5, human and macaque PBMCs). Similarly, the HIVenv viruses were produced through co-transfection of 293T cells with pREC_nfl_HIV_env_X and the complementary vector, pCMV_cplt [20]. The supernatants from transfected 293T were added to U87.CD4.CCR5 cells to produce infectious virus as described [20]. Virus production was monitored in the culture fluids using a reverse transcriptase (RT) assay as described [22]. SHIV p27 capsid levels in the supernatants of transfected 293T cells or infected cell lines was also measured using the RETRO-TEK SIV p27 Antigen ELISA assay (ZeptoMetrix Corporation) according to the manufacturer protocol.

### Virtual and actual TCID_50_ determinations

Since the SHIVenv viruses showed limited replication in primary cells and cell lines, these virus titers were estimated from RT activity using “Virtual TCID_50_ assay” as previously described [22]. Briefly, serial dilutions (1:4) of each HIV-1env and SHIVenv chimeric virus were performed in quadruplicate and then used (1) to infect U87.CD4.CCR5 cells (only with HIV-1env) or (2) to determine the RT activity in each dilution. There was nearly a perfect correlation (r > 0.95, p < 0.001; Pearson product moment correlation) between HIV-1env infectious titers (as determined by an actual TCID_50_ assay) and the virtual titers measured by RT activity. Thus, the SHIVenv titers were estimated from the RT activity in the serial dilutions of the virus-containing 293T supernatants.

### Veritrop assay

The function of the HIV-1 *env* insert within the HIV-1 or SHIV_KB9_ backbone was analyzed using a cell-to-cell fusion assay, Veritrop as described [23]. Approximately 5 × 10^5^ U87.CD4.CCR5 cells were transfected with pDM128fLuc using the FuGENE 6 Transfection Reagent (Roche) according to the protocol provided by manufacturer. Concurrently, 293T cells (5–6 × 10^4^ cells per well) were transfected with pREC_nfl_SHIVenv_X_ vector. After 24 h (on day 2), the pREC_nfl_SHIVenv_X transfected 293T cells were layered on top of the pDM128fLuc-transfected U87.CD4.CCR5 cells (5–6 × 10^4^ cells per well) in a 24 well plate. At 48 h, cells were harvested and lysed with 100 μl of Glo Lysis Buffer and combined with 50 μl of Bright Glo (Promega Biotech), then read with Victor^3^ V (Perkin Elmer) for luciferase activity.

### Affinofile assay

Affinofile cells were employed to examine CD4 and CCR5 affinity/avidity during virus entry as previously described [24, 25]. Affinofile cells were plated at a density of 10,000 cells per well in a 96-well plate and then induced to express high to low levels of CD4 with minocycline (5–0 ng/ml, twofold dilutions over six separate dilutions) [25] and high and low CCR5 expression with ponasterone A (4 and 0.125 μM) as described [24, 25]. Cells were induced for 24 h prior to infection with HIV-1 pseudotyped with the various AHI *env* genes [24, 25] for 48 h, washed with PBS, and lysed with Glo lysis buffer (Promega, Inc.). Maximal infection was considered luciferase activity generated by infection at the highest CD4 and highest CCR5 concentration. CCR5 and CXCR4 expression levels per cell was measured at 24 h (prior to infection) by flow cytometry as described [24, 25].

### HIV-1 competitions for replicative fitness analyses

Dual virus infections were performed in PHA-activated, IL2-treated peripheral blood mononuclear cells of HIV-negative donors as described [26]. Each HIV-1env chimeric virus derived from an AHI was competed against three competitor viruses, i.e. HIV-1env chimeric viruses derived from chronic subtype B infections (Q0, I10, and K44). Dual infections involved both AHI and reference viruses added to PBMCs at 0.005 multiplicity of infection (MOI). Overall virus production was monitored by RT activity. Relative virus production and fitness was analyzed by the classical heteroduplex tracking assay described in [26, 27] and by using an amplicon-based 454 pyrosequencing approach (described below).

### 454 pyrosequencing

Genomic DNA from dually infected cells was isolated using the QIAamp 96 DNA Blood Kit. PCR primers were designed for Roche GS Flx System and modeled after the E110-E125 primer pairs (sequence: 5′-CTGTTAAATGGCAGTCTAGCAGAA, and 5′-CAATTTCTGGGTCCCCTCCTGAGG) where the E110 primer (i.e. Roche GS FLX A and B primers) contained a five base-pair barcode (GCCTCCCTCGCGCCAXXXXX) and the E125 primer represented the Roche GS FLX B primer. The 250 E110 primers were designed with distinct barcodes and to be paired with E125. Using two HIV-1 DNA templates, PCR with each primer pair resulted in equal amplification efficiency and the same size DNA product (357 bp). One DNA sample from a single competition was PCR amplified with one designated primer pair. Please note, in order to eliminate the possible PCR-induced recombinants, we used Phusion High-Fidelity DNA Polymerase (NEB) which can rapidly complete the DNA extension (15–30 s per Kb) and longer extension time (2 min). PCR products were purified using the QIAquick 96 PCR Purification Kit and quantified using Quant-iT PicoGreen dsDNA Assay Kit (Invitrogen), and then equalized to a concentration of 10 ng/μl. Samples were pooled into two tubes to prevent redundancy of barcoded primer used for the amplification of samples. We used GS emPCR Kit II (Roche) to obtain reads starting from Primer A. Amplicons were added to the emulsion at one copy per bead. EmPCR was done according to the Roche EmPCR manual, and Amplicon sequencing was performed according to the Roche Amplicon sequencing manual. Sequences were processed and collected using Roche GS FLX System software. Sequences were extracted according to barcode by the Java script Extraction (written by John Archer) and sorted by barcode into a folder containing to different files (raw sequence, aligned sequence, and predicted amino acid sequence) related to specific dual virus competitions. The extracted sequences were then analyzed using the Competition Table java script, which aligns and counts specific sequences derived from 454 analyses (and with a specific barcode) using the known reference sequences. Additionally, phylogenetic trees were generated for ~20% of the competitions to verify the results from the Competition Table java script. It is important to stress that any errors generated by 454, as we have previously described [28], do not impact the determination of replicative fitness. Competition Table java script aligns to one of two HIV-1 sequences in the competition (based on the input sequences of these AHI env clones) such that any single and multiple point mutations within a viral quasispecies or generated by PCR/454 error still aligns to one of two diverse HIV-1 (found in the competition). Production of each HIV-1 isolate (as determined by sequence count) in a dual infection (*f*_0_) was divided by the initial proportion in the inoculum (*i*_0_) to determine the relative fitness (*W* = *f*_0_ */i*_0_). Fitness difference (*W*_*D*_) is the ratio of the relative fitness values of each HIV-1 isolate in the competition (*W*_*D*_ = *w*_*M*_ */w*_*L*_) [26, 27]. Finally, the relative fitness values in a competition derived by 454 sequence analyses showed less than 10% variance with those derived by HTA analyses (data not shown).

## Results

### Construction of yeast-based cloning vectors for producing SHIVenv viruses

We previously constructed pREC_nfl_HIV and pCMV_cplt vectors for yeast-based cloning of diverse HIV-1 strains [20]. As described in Additional file 1: Figure S1, we developed a similar method for SIV and SHIV cloning. The 5′ half of HIV genome in pREC_nfl_HIV vector was first replaced with URA3 then substituted with the 5′ half of the SHIV_KB9_ genome. The 3′ half of SHIV_KB9_ was introduced using the same procedure to form the vector of pREC_nfl_SHIV_KB9_; this vector contains the near full length of SHIV_KB9_ genome and lacks the 5′ repeat (R) and unique (U5) regions. The complementing vector, pREC_SHIV_KB9__cplt_R/U5/gag was constructed by swapping the 5′LTR/gag sequence of HIV-1 with that of SHIV_KB9_ as described (Additional file 1: Figure S1).

To test this SHIV_KB9_ vector system, pREC_nfl_SHIV_KB9_ and the complementing vector, pREC_cplt_R/U5/gag, were co-transfected into 293T cells to produce SHIV_KB9_ virus, which was then used to infect 174×CEM.CCR5 cells, a human B&T cell hybrid line. The parent virus, SHIV_KB9_, produced by 293T co-transfections, could be propagated in 174×CEM.CCR5 cells as previously reported [29] but virus production (12 days post-infection) was delayed when compared to that of HIV-1_NL4-3_ (3 days post-infection) (Additional file 2: Figure S2 A). This delay in virus outgrowth is consistent with previous studies of SHIV_KB9_ virus propagation [30]. SHIV_KB9_ could infect and replicate in U87.CD4.CCR5 cell cultures but only after initial propagation on 174×CEM.CCR5 cells whereas SHIV_KB9_ derived from the transfected 293T supernatants did not expand in U87.CD4.CCR5 cells (Additional file 2: Figure S2 B).

**Fig. 1:**
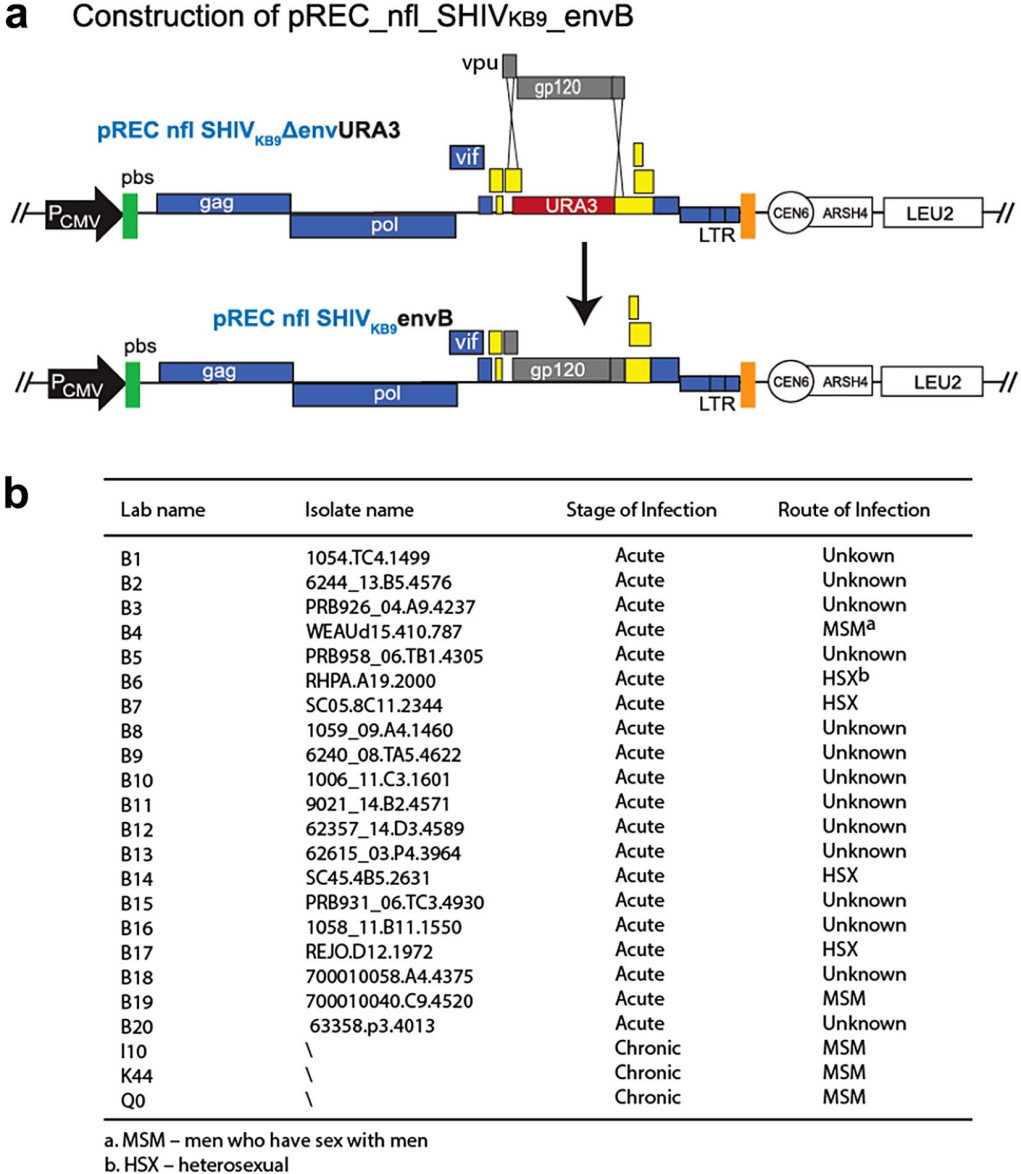
Production of pREC_nfl_SHIVenv containing the gp120 coding region derived from acute HIV-1 infections. **a** Construction of pREC_nfl_SHIVKB9_envB vector/URA3. The *URA3* gene and the corresponding AHI *env* B genes only replace a region flanked by KB9 sequence (HXB2 numbering nt5996-nt8638). SHIV_KB9_ is a SIVmac239 virus containing only a portion of the HIV-1 env_89.6_ gene, i.e. from the 1st exon of tat to the end on the 2nd exon of tat (HXB2 numbering nt5823-nt8676, shadowed in *yellow*). **b** 20 acute HIV-1 subtype B envelope (Env) and three chronic HIV-1 Envs (i.e. I10, K44, and Q0) were cloned into pREC_nfl_SHIV_KB9_Δenv/URA3 (or pREC_nfl_HIVΔenv/URA3) by homologous yeast recombination/gap repair to produce the pREC_nfl_SHIV_KB9__env_B1-20_ and pREC_nfl_HIVenv_B1-20 vectors

To produce the SHIVenv chimeric virus, we first constructed pREC_nfl_SHIV_KB9__Δenv/URA3 vector in which SHIV_KB9_ *env* was replaced with the *URA3* gene of yeast. It is also important to stress that SHIV_KB9_ is a SIVmac239 virus containing only a portion of the HIV-1 env_89.6_ gene, i.e. from the 1st exon of *tat* to the end of the 2nd exon of *tat* (HXB2 numbering nt5823-nt8676). The *URA3* gene replaced only the HIV-1 sequences in KB9 (HXB2 numbering nt5996-nt8638) to generate the pREC_nfl_SHIV_KB9__Δenv/URA3 cloning vector (Fig. 1).

### SHIVenv chimeric virus production

**Fig. 2:**
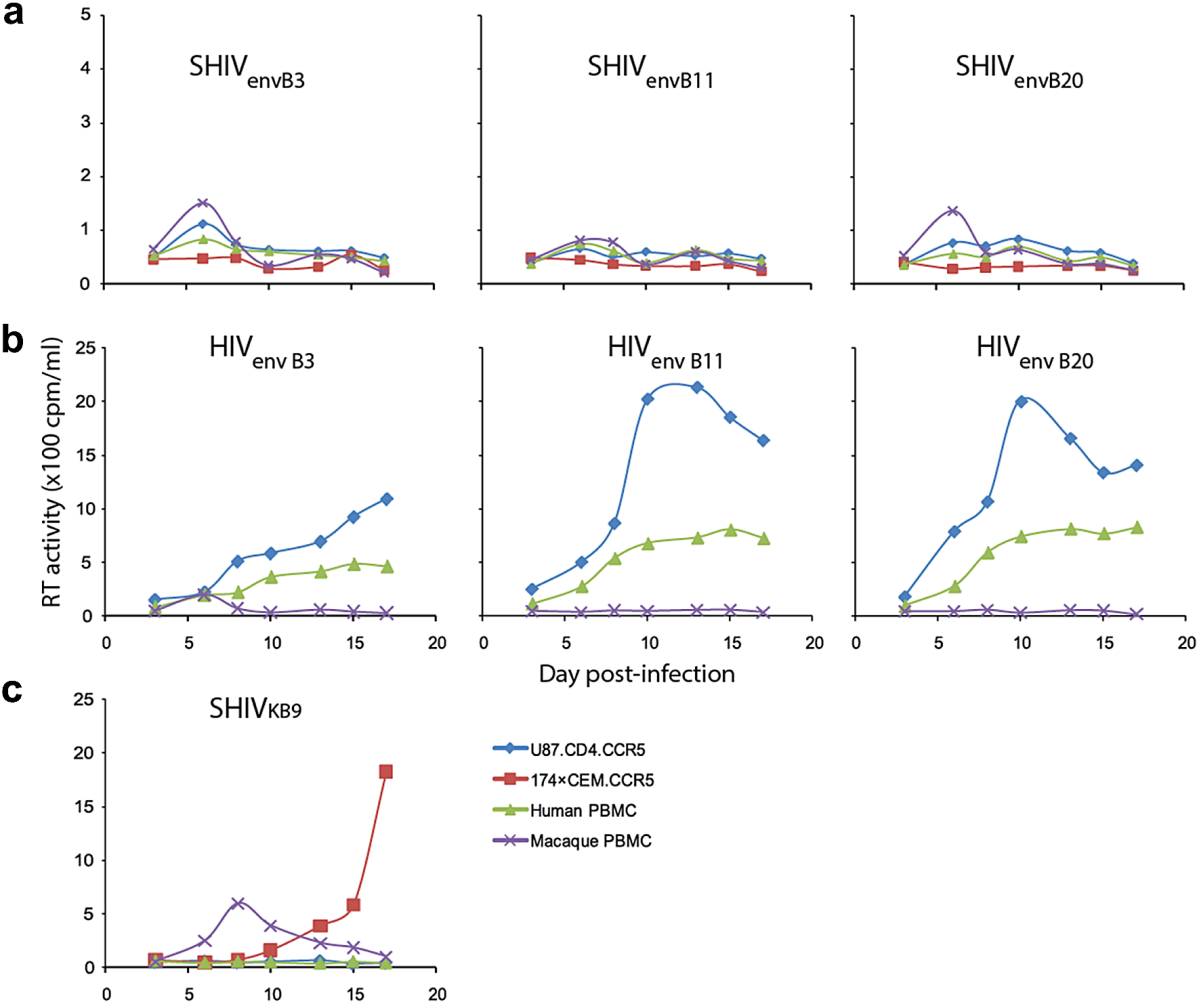
Chimeric viruses SHIVenv_B and HIVenv_B propagation in different PBMCs and cell lines. U87.CD4.CCR5, 174×CEM.CCR5, human PBMCs or macaque PBMCs were exposed to SHIVenv_B3, _B11, and _B20 (**a**), or HIVenv_B3, _B11, and _B20 (**b**), or SHIVenv_KB9 (**c**) chimeric viruses. Virus production was measure by RT activity in the supernatant over 17 days

Twenty PCR-amplified subtype B *env* clones (B1 to B20; Fig. 1a), isolated from acute HIV infections, were cloned into the pREC_nfl_SHIV_KB9__Δenv/URA3 vector via yeast recombination/gap repair [20] (Fig. 1b). The pREC_nfl_SHIVenv_B1-to-B20 vectors were then co-transfected with the complementary vector pREC_cplt_R/U5/gag into 293T cells to produce SHIV_KB9_env_B1 to SHIV _KB9_env_B20 viruses (designated as SHIVenv_BX for simplicity thereafter), which were then tested for infectivity in different cell lines (i.e. U87.CD4.CCR5 and 174×CEM.CCR5), human and macaque PBMCs. Only transient replication over 5–7 days was observed with the 20 SHIVenv_B1 to _B20 viruses in the various cell lines, macaque, and human primary cells. The SHIV with the AHI *env*s could not be propagated or expanded, while the parent SHIV_KB9_ virus was efficiently propagated in both macaque PBMCs and 174×CEM.CCR5 cells (Fig. 2a, c). The same cloning and virus production procedure were employed to produce the HIV-1env_B1 to _B20 chimeric viruses (the counterpart to the SHIVenv_B1 to _B20). In contrast to the SHIVenv viruses, all of the HIV-1env_BX chimeric viruses containing the subtype B AHI envelope in an NL4-3 backbone were infectious and were passaged through a susceptible cell line (i.e. U87.CD4.CCR5) and human PBMCs, but not macaque PBMCs (Fig. 2b).

### Testing of the function of HIV-1 envelope proteins in SHIV_KB9_ backbone

**Fig. 3:**
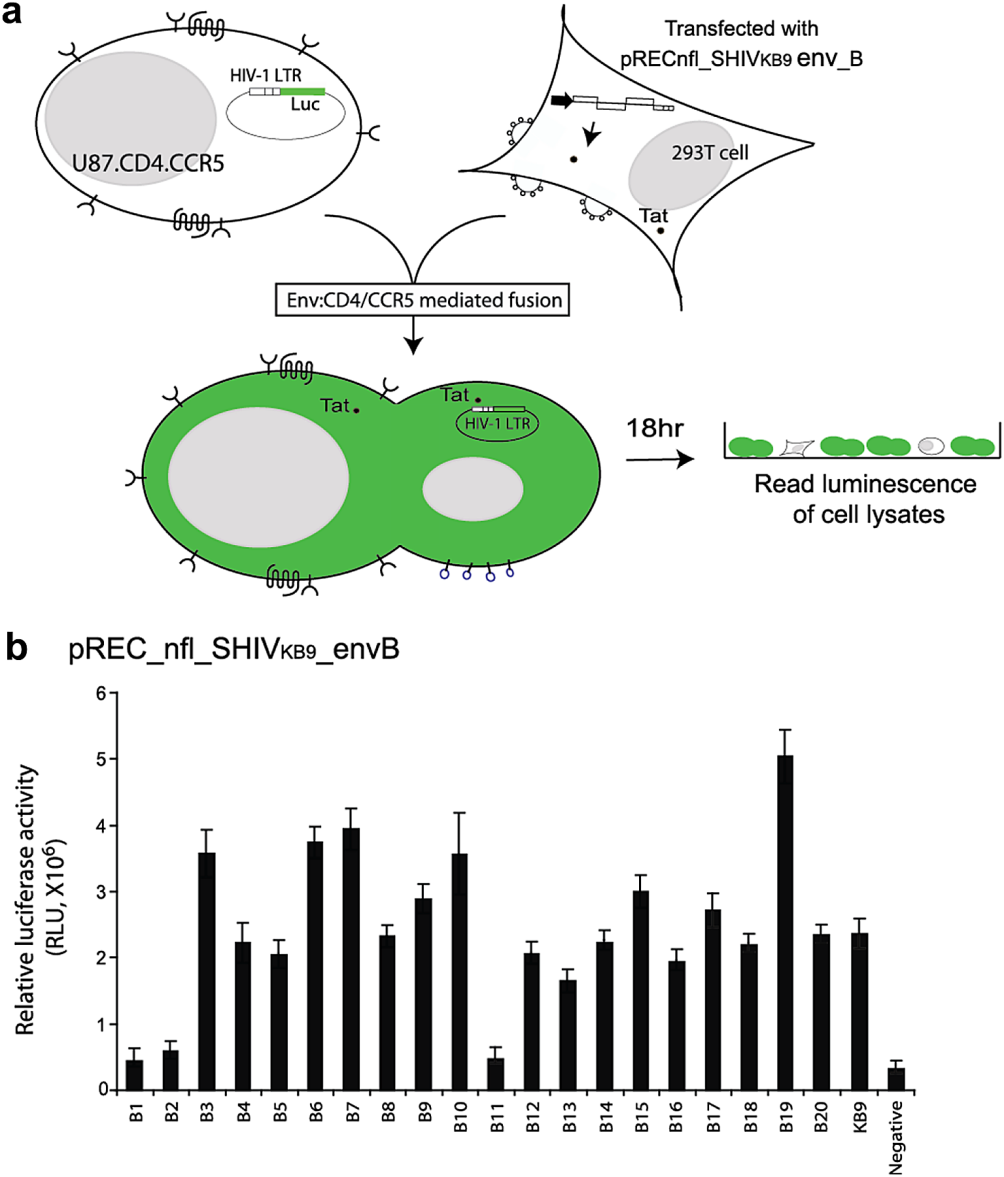
Veritrop assay detecting the function of the HIV-1 envelope proteins in the context of SHIV_KB9_ backbone. A schematic of the Veritrop cell-to-cell fusion assay is shown in (**a**). The target cell, U87.CD4.CCR5 was transfected with pDM128fLuc, a vector where expression of firefly luciferase gene is dependent on both HIV-1 Rev and Tat produced by the effector cell [34]. The effector cell is transfected with pREC_nfl_SHIV_KB9_env_B vectors, which express entire SHIV proteome and produce virus particles that lack the proper SHIV genomic RNA for de novo replication [20]. Levels of cell fusion (via relative light units from Luciferase activity) are shown for the effector cells transfected with pREC_nfl_SHIV _KB9_env_B vectors (**b**)

Inability of the SHIVenv_B1 to _B20 chimeric viruses to propagate in cell lines and primary cells could be related to incompatibility between the HIV-1 insert and the SHIV_KB9_ backbone. It is important to note that SHIV_KB9_ backbone contains an HIV-1 KB9 *env* gene that extends from the beginning of tat1 to the end of tat2/rev2. All of our env_B cassettes were placed within the KB9 sequence (see “Methods” section). The function of the HIV-1 envelope protein in the context of SHIV_KB9_ backbone was tested using the Veritrop Assay, a cell-to-cell fusion assay. 293T cells were transfected with the pREC_nfl_SHIV_KB9_env_B vectors and then layered over U87.CD4.CCR5 cells transfected with vector expressing firefly luciferase under the control of both HIV-1 Tat and Rev (Fig. 3a). Unlike other cell-to-cell fusion systems, our pREC_nfl vectors express all of the viral proteins, in the correct stoichiometry, and with the ability to form virus particles [20]. However, in the absence of the complementing vector, these virus particles are non-infectious. We recently discovered that our cell fusion assays (Veritrop) is approximately 100-fold more efficient than cell fusion system mediated by just an Env expression vector (R. Gibson, E. J. Arts, D. McDonald, et al. manuscript in preparation).

Using our Veritrop assays, the majority of Env glycoproteins derived from pREC_nfl_SHIV_KB9_env_B were capable of binding to the CD4 and CCR5 receptors and mediating cell fusion (Fig. 3b). Only 3/20 SHIVenv_B vectors (i.e. B1, B2, and B11) did not mediate sufficient cell-to-cell fusion to justify inclusion in the SHIVenv_B pools for future macaque infection studies (see below). Interestingly, dysfunction did not appear to be related to the AHI sequence since (1) all the B clones were functional in the HIV-1_NL4-3_ backbone and (2) the AHI *env* sequences were in the correct reading frame with the flanking KB9 *env* sequences. This data suggest that the function of these Env glycoproteins may be impacted by poor complementation in the KB9 backbone. We did confirm gp120 expression by Western blot for a subset of the pREC_nfl_SHIV_KB9_env_B and pREC_nfl_HIV-1_NL4-3_env_B expression vectors transfected into 293T cells (data not shown).

### Testing of the expression level of P27 and reverse transcriptase in SHIVenv_B chimeric viruses

**Fig. 4:**
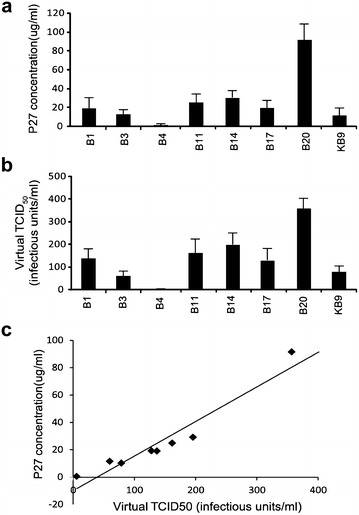
Expression of p27 capsid and reverse transcriptase of the SHIVenv_B in the transfection supernatant from 293T cells. SIV p27 was monitored by antigen capture assay in the supernatant of 293T cells transfected with pREC_SHIV_KB9_env_B (B1, B3, B4, B11, B14, B17 and B20) (**a**). Reverse transcriptase activity was also measured using serial dilutions of the supernatant from the same transfected cells. RT activity is report as virtual TCID50 as previously described (**b**) [22]. The correlation of p27 and virtual TCID50 of each SHIVenv_B virus was plotted (**c**)

The majority of the SHIV_KB9_env_B constructs could express functional HIV-1 envelope glycoproteins and yet, these viruses showed limited replication in different cell lines or macaque PBMCs. However, it is also important to note that SHIVenv and some SIV strains commonly show poor replication efficiency in cell culture despite the ability to infect macaques [13, 30, 31]. For a crude assessment of the virus particles, expression of p27 capsid levels and reverse transcriptase activity was measured in the SHIVenv_B derived from supernatants of transfected 293T cells. SHIVenv_B1, _B3, _B11, _B14, _B17, and _B20 (except SHIVenv_B4_) had similar or higher levels of p27 capsid protein and RT activity than SHIV_KB9_ (Fig. 4a). The ratio of p27 capsid to RT activity was similar for all of these SHIVenv_B viruses (Fig. 4a–c).

### SHIVenv infection in rhesus macaques

**Fig. 5:**
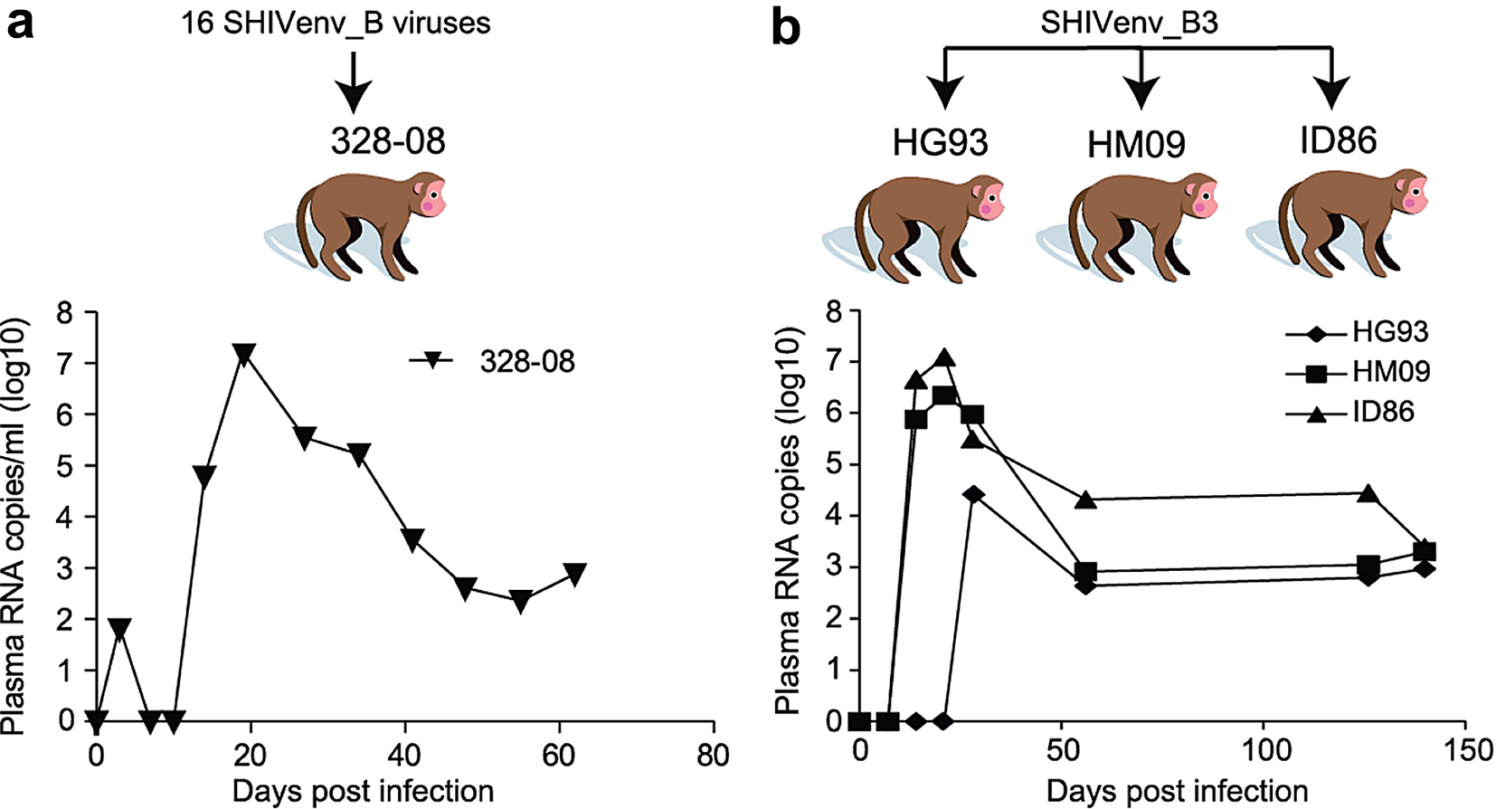
SHIV replication in macaques exposed to a pool of 16 SHIVenv_B viruses or just SHIVenv_B3. **a** The SHIVenv_B pool harboring 16 chimeric viruses were intravenously injected into two macaques of Indian origin. Infection of the two macaques was monitored by collecting plasma samples for 62 days and measuring viral RNA levels by quantitative real time RT-PCR for SIV Gag (Duke Human Vaccine Institute). Results are only shown for animal m328-08 in which SHIVenv infection was established. **b** The SHIVenv_B3 was used to infect three other rhesus macaques of Indian origin. Viral RNA was monitored for 140 days

Following the assessment of p27 capsid levels, RT activity, and envelope glycoprotein function, we combined ~100 IU of 16 SHIVenv_B_ (i.e. B3, B5-B10, B12-B20, ~1600 IU in total). Two animals (m328-08) were then intravenously injected with the SHIVenv_B pool (Fig. 5a). One macaque (i.e. 328-08) was infected showing peak viremia of 10^7^ copies/ml at 3 weeks post exposure. The infected 328-08 macaque supported sustained viral replication over a period of 3 months even though the viral level decreased to 10^3^ copies/ml by day 40 post-infection.

**Fig. 6:**
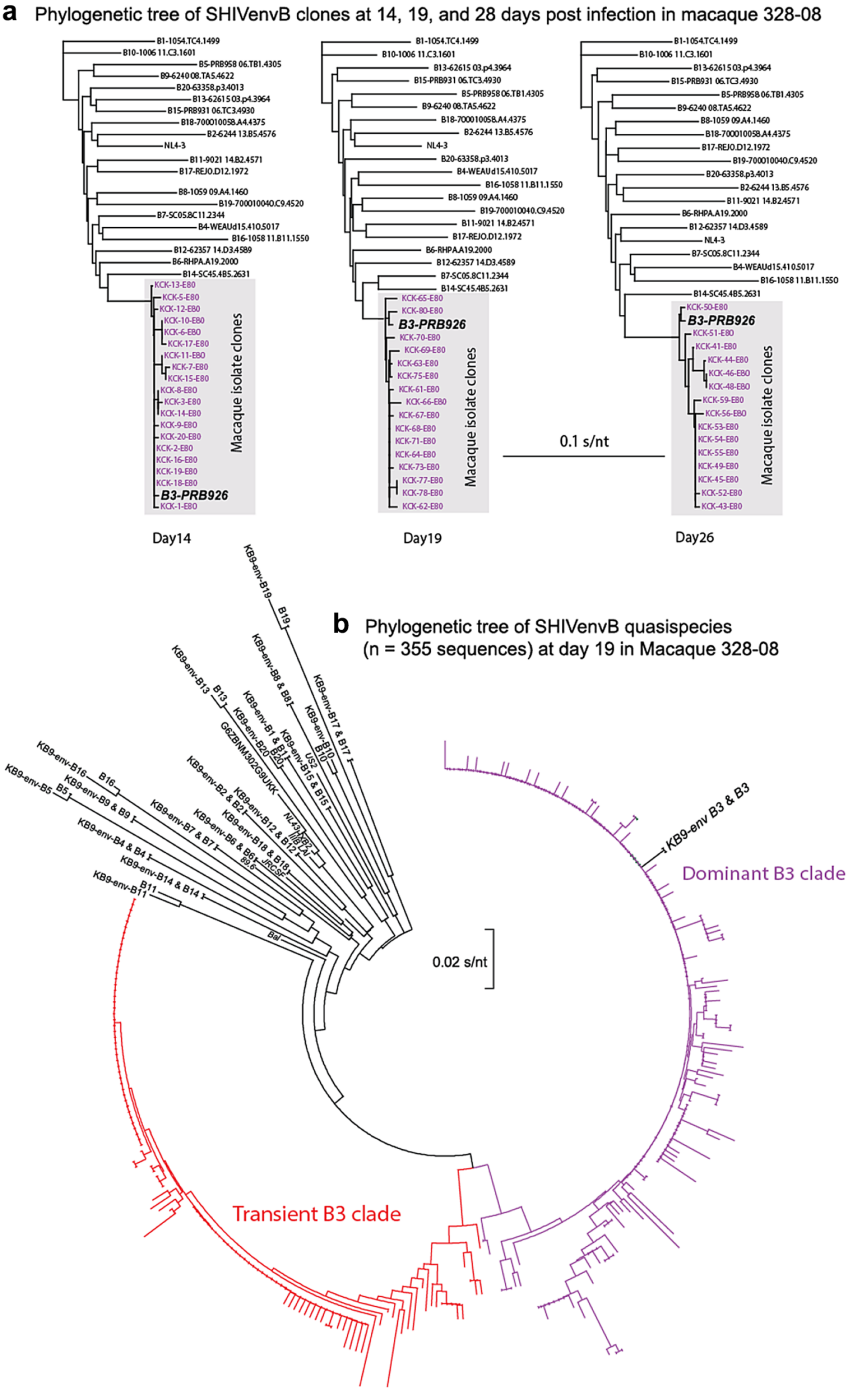
Sequence analyses from plasma of the infected macaques. HIV RNA was extracted from plasma collected from the m328-08 and then subjected to RT-PCR amplification, TOPO cloning, and sequence analyses of 15–19 clones from days 14, 19, and 26 post inoculation. A 480 nt gp120 coding region (C2-V3) of the m328-08 clones were aligned with all the SHIVenv_B clones within the inoculating pool using CluxtalX. Neighbor joining trees were constructed using FigTree and presented in (**a**). Only a single SHIVenv_B3 clone established and maintained infection in macaque 328-08. The RT-PCR amplified HIV-1 gp120 product derived from ~10,000 RNA copies at day 19 was subjected to 454 pyrosequencing. The C2-V3 sequences from day 19, the 16 SHIVenv___B sequences from the inoculating pool, and a set of subtype B reference sequences were aligned using Bayesian algorithm (Beast) [73] and trees were constructed using a maximum likelihood method. The tree presented in **b** was drawn with FigTree v1.4 (http://tree.bio.ed.ac.uk/software/figtree)

Plasma samples were collected from m328-08 at various time points and RT-PCR amplified for the SHIV *env* DNA. We performed clonal sequencing and 454 pyrosequencing of the C2-V3 region at days 14, 19 and 26 of m328-08. Both Sanger sequencing of at least 15 clones from each time point (Fig. 6a) and 454 pyrosequencing at read depth of ~1000 sequences/sample revealed that only a single clone (i.e. B3-PRB926) in the pool of 16 SHIVenv_B chimeric viruses established infection in m328-08. The average read length of ~500 nt for 454 pyrosequencing was trimmed to 300 nt, aligned using Beast, and presented on the phylogenetic tree in Fig. 6b. Although all sequences in all time points aligned with a SHIVenv_B3, the virus at d19 in m328-08 had two distinct B3 clades at nearly equal proportions. By day 26, the more divergent B3 clade could be identified. The second clade could be related to a PCR and/or sequencing error but this appears unlikely considering that at least 5–10 nt substitutions separate the two clades. The most obvious possibility is a recombination between B3 and one or more of other 15 SHIVenv_B viruses in the pool. However, preliminary analyses using Frag-Dist [32] could not identify simple breakpoints and regions of homology between B3 and other 15 env_B clones. More thorough analyses using program such SplitTree [33] are ongoing.

Based the preferential infection of SHIVenv_B3 over the other 15 SHIVenv_B viruses, we exposed another three macaques to this virus pool but again failed to establish infection. However, three macaques were infected by an inoculum containing just SHIVenv_B3 virus (100 IUs) (Fig. 5b). These three macaques infected with SHIVenv_B3 virus reached peak viremia (10^4.4–7.1^ copies/ml) at day 21 or day 28 and the viral level still maintained around 10^3^ copies/ml by day 140. It is important to note that SHIVenv_B3 virus had similar p27 capsid levels, RT activity, and Env function as the other 15 SHIVenv_B viruses. None of the SHIVenv_B viruses derived from AHI could propagate in cell lines or primary PBMCs.

### Sensitivity to entry inhibitors, kinetics and efficiencies of host cell entry

**Fig. 7:**
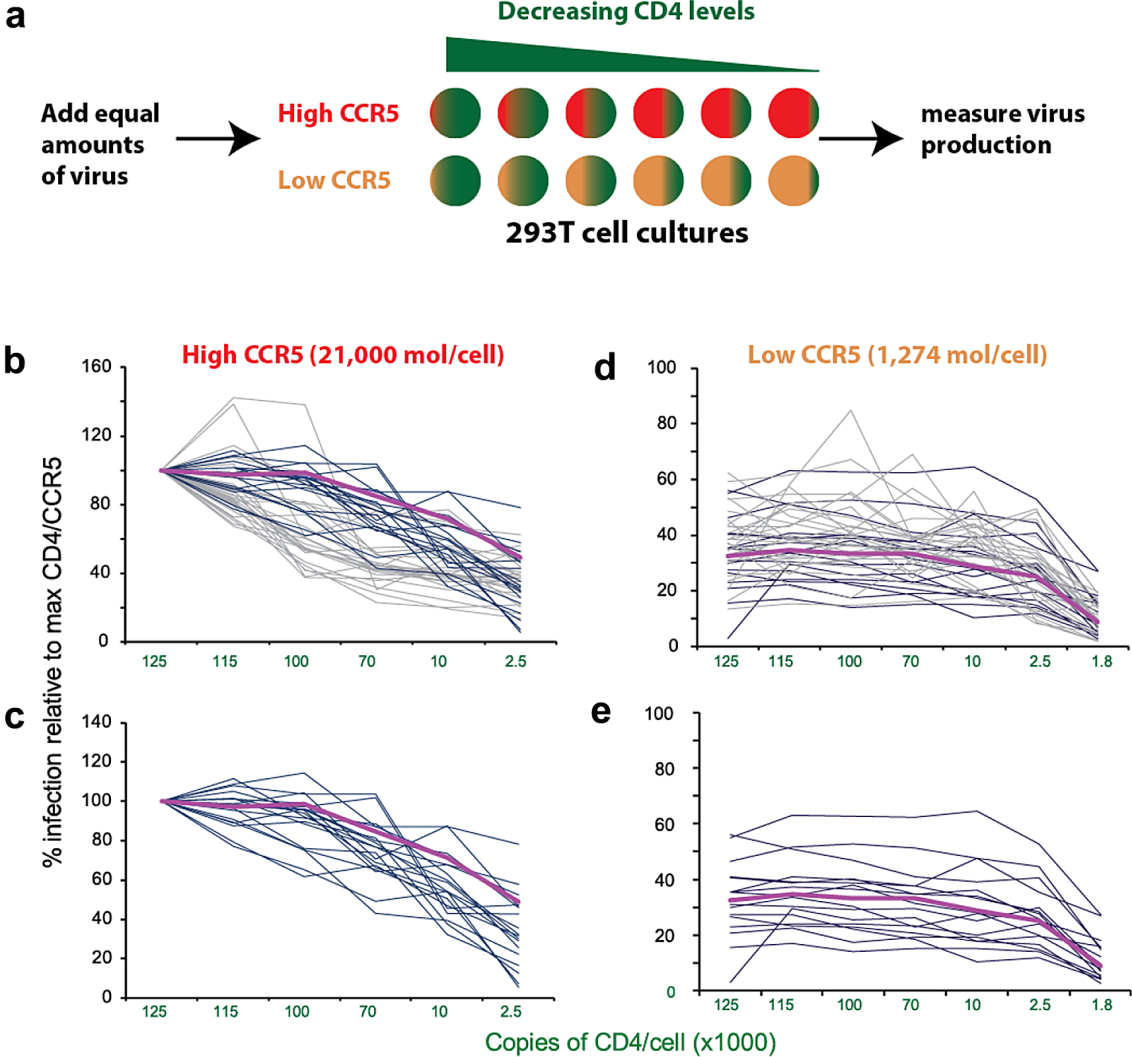
Using the Affinofile system to measure entry efficiency of the HIVenv_B chimeric viruses. **a** The 293-Affinofile cells were treated with ponasterone A (4 and 0.125 ng/ml) to obtain high (~15,830 copies/cell) and low CCR5 surface expression (~1365 copies/cell) and with increasing concentrations of minocycline to obtain range of CD4 surface expression (1800–125,000 copies/cell). 293T cells with different CD4/CCR5 levels were exposed to each of the Env pseudotyped viruses [25] using the Env expression plasmids derived the CHAVI AHI [7] (**b**, **c**) and the NYU/Aaron Diamond AHI cohort [25] (**d**, **e**). Virus production was monitored by luciferase expression from the LucAM transgene [25]. An average maximal infection was calculated from the relative light units (RLUs) produced from infection of 293T with the highest CD4 and CCR5 surface expression. All other levels of infectivity are plotted as a percentage of the RLU obtained relative to RLU with maximal CD4 and CCR5 expression. **b**, **c** Indicate levels of infectivity at high CCR5 over a range of CD4 and **d**, **e**, low CCR5 over a range of CD4. The level of infectivity was measured and presented for all HIV-1env clones derived from AHI (**b**), only those HIV-1env clones from CHAVI AHI patients which were also used to construct the SHIVenv pool (**c**). In all four graphs, the level of infectivity with the HIV-1env B3 clone (i.e. the SHIVenv_B clone establishing infection in the macaque) is indicated with *purple line*. The *dark blue* and *light blue lines* are the levels of infectivity using the AHI HIV-1env clones from CHAVI and the NYU/Aaron Diamond cohort, respectively

Since SHIVenv viruses cannot maintain virus replication in culture, we cloned the identical *env* cassettes into HIV-1 vectors for subsequent studies characterizing the “transmission” phenotype of the Env_B3 versus other Env’s derived from AHI. We used the Affinofile system to measure the efficiency of HIV-1 entry into host cells expressing a range of physiological CCR5 and CD4 receptor concentrations on the cell surface. As previously described, greatest dynamic range in HIV-1 entry was observed with low and high levels of CCR5 (~1400 and 16,000 per cell) with varying levels of CD4 receptor (2500–125,000 copies/cell) [24, 25] (Fig. 7). Twelve wells with these CD4/CCR5 levels on 293T cells were exposed to each of the 20 HIV-1env_B viruses and 38 other HIV-1env chimeric viruses from the NYU/Aaron Diamond AHI cohort. Virus entry was monitored by Rev/Tat induction of the luciferase expression (see “Methods”). As described in Fig. 7b–e, we could not distinguish significant differences between any of the 20 HIV-1env chimeric viruses in terms of utilizing CD4 and CCR5 for host cell entry. The HIV-1env_B3 virus appears slightly more efficient at virus entry with high CCR5 levels across a range of CD4 when compared to other AHI chimeric env viruses. Nonetheless, these differences between B3 versus and other HIV-1env_B chimeric viruses were not significant based on the triplicate analyses. This slight advantage in entry efficiency by the B3 virus was not observed at low CCR5 levels.

**Fig. 8:**
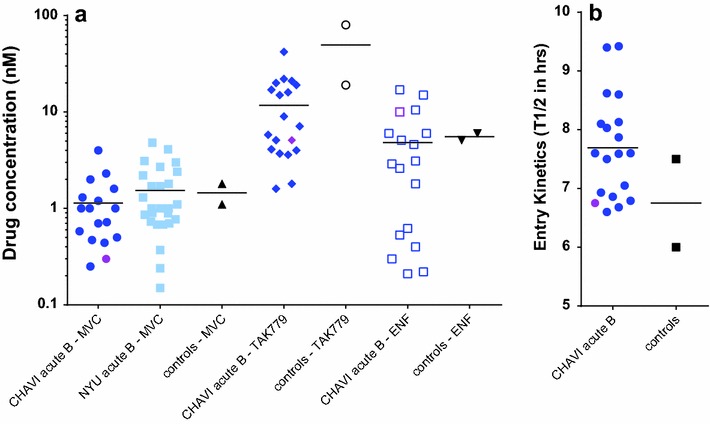
Sensitivity to entry inhibitors and entry kinetics. **a** Each HIV-1env___B virus (CHAVI acute, NYU acute, and two chronic HIV-1 strains as control) was tested for inhibition by enfuvirtide, TAK779, and maraviroc. Inhibitory concentration for 50% inhibition (IC_50_) was plotted for AHI HIV-1env viruses in **a**. The IC_50_ values of HIV-1env_B3 are shown with *purple symbols*. **b** Entry kinetics was measured with the HIV-1env_B samples by performing time-of-drug inhibition experiments with the RT inhibitor, 3TC

Sensitivity to entry inhibitors is another method to detect phenotypic differences between HIV-1env chimeric viruses. Each HIV-1env_B virus was tested for inhibition by enfivuritide (ENF; a fusion inhibitor), TAK779, and maraviroc (MVC; CCR5 antagonists). Inhibitory concentrations for 50% inhibition (IC_50_) were plotted for each virus in Fig. 8a. Overall, all HIV-1env_B viruses including HIV-1env_B3 had similar IC_50_ values to ENF, TAK779, and MVC. HIV-1env_B3 may be slight more sensitive to MVC inhibition but again this virus was not an outlier when compared to range in MVC IC_50_ values with HIV-1env chimeric viruses derived from NYU/ADARC AHI samples.

Finally, entry kinetics was measured with the HIV-1env_B samples by performing time-of-drug inhibition experiments. Various entry inhibitors (soluble CD4, MVC/TAK779, or ENF) block different steps but not the entire host cell entry process and as such, we examined the timing of complete virus entry using a nucleoside RT inhibitor (lamivudine) to block the subsequent step in the virus life cycle, i.e. reverse transcription. In addition, the RT coding region is isogenic in this chimeric virus and any increase or decrease in the time window of 3TC inhibition likely reflects differences in the preceding entry step and not in the reverse transcription step. When comparing the T_1/2_ for 3TC among the HIV-1env chimeric viruses, there was ~2 h range in the time required for entry which is consistent with previous studies [25, 34]. The predicted time for HIV-1env_B3 entry into host cells falls within the time range for entry of the other HIV-1env_B chimeric viruses (Fig. 8b). Based on entry efficiency/receptor utilization, sensitivity to entry inhibitors, and entry kinetics, there was no statistical evidence to suggest that Env_B3 glycoprotein was better than the other Env_B glycoprotein in the HIV-1 backbone. However, the trend for increased Env_B3 function in all three activities might suggest a collective fitness advantage of SHIVenv_B3 over the other SHIVenv_B’s in the inoculating pool.

### Replicative fitness of the HIV-1env chimeric viruses

**Fig. 9:**
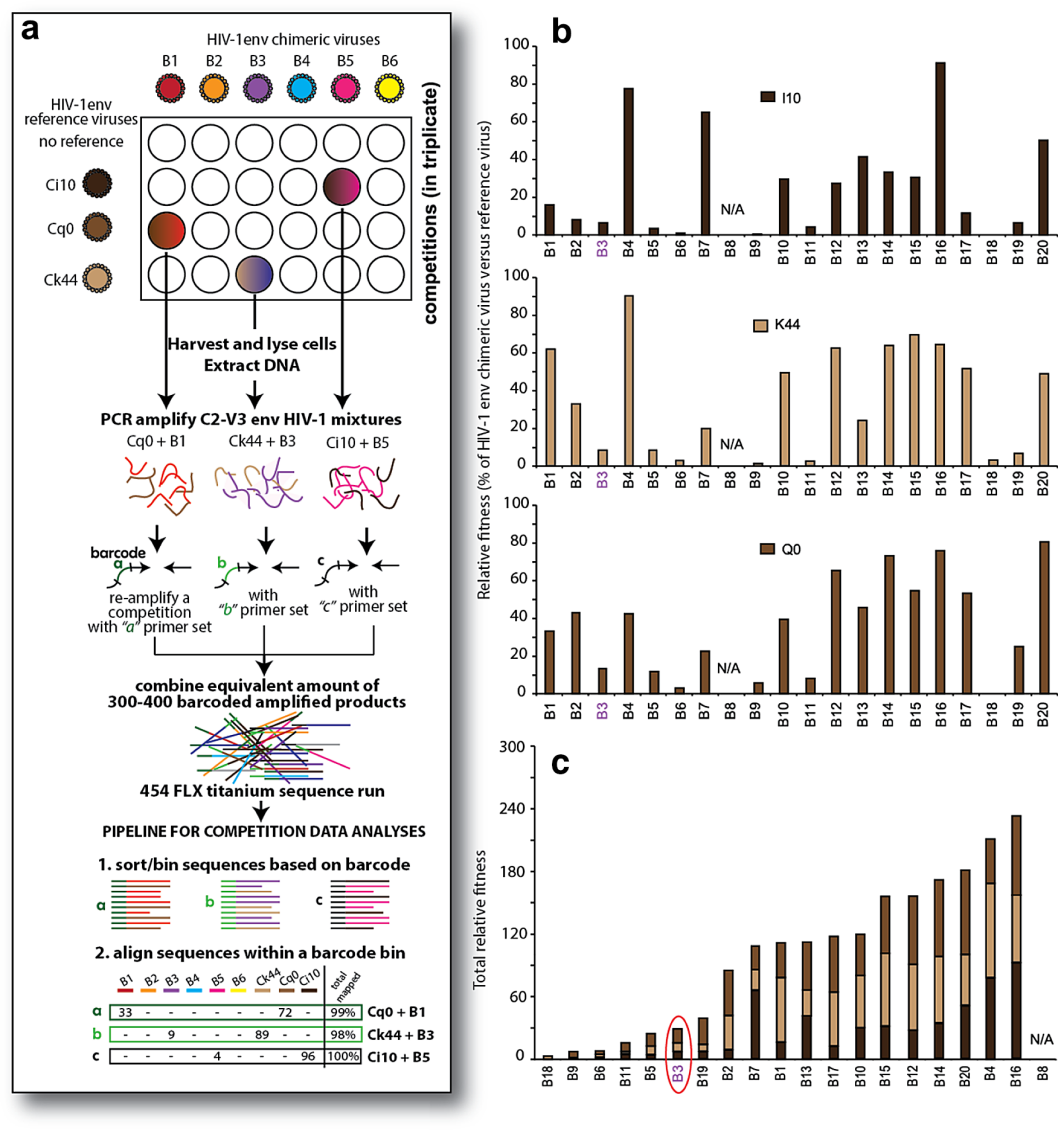
The fitness difference of AHI HIV-1env chimeric virus relative to the control HIV-1env chimeric virus derived from chronic infection. As described in the schematic (**a**), HIV-1env_B chimeric viruses were competed against three reference viruses (i.e. HIVenv_I10, HIVenv_K44, and HIVenv_Q0) in HIV-negative PBMCs as described in the “Methods” section. **a** is also schematic describing the PCR and 454 pyrosequencing methods used to detect and quantify dual infection (see “Methods” section for more detail). The percent replication relative to HIVenv_I10, HIVenv_K44, and HIVenv_Q0 is shown in **b**. Total relative fitness (the addition of all relative fitness values) is presented in **c** to provide a rank order for relative fitness for the 19 HIVenv viruses tested

Receptor binding, entry efficiency and kinetics are only one set of factors in determining replicative fitness. However, with Env chimeric viruses and isogenic backbone, we have reported that replicative fitness maps to the *env* gene and is typically dominated by entry efficiency [27, 35, 36]. To investigate the fitness differences among these 20 AHI envelope clones, HIV-1env chimeric viruses were competed against three subtype B reference viruses (i.e. HIVenv_I10, HIVenv_K44, and HIVenv_Q0) in HIV-negative PBMCs (Fig. 9a). Following 10 days of dual infection, a segment of the HIV-1 *env* gene was PCR amplified with primers containing barcodes for 454 amplicon pyrosequencing. Each barcoded “bin” of sequences were aligned to the two viral sequences in the competition and counted to determine the relative replicative fitness values in each dual infection (Fig. 9a). Details of this method and comparisons to other classical approaches to measure dual virus production were described in [26, 27]). Based on these fitness analyses, B16, B4 and B20 in HIV-1_NL4-3_ backbones had highest replicative fitness when competed against the three subtype B reference viruses (Fig. 9c). In these analyses, HIV-1env_B3 was outcompeted by all three reference strains and was in the lower range of replicative fitness when compared to the other HIV-1env_B chimeric viruses. In fact, HIV-1env_B3 ranked 14th in relative replicative fitness out of the 19 HIV-1env_B viruses (B8 failed for the detection) and 11th out of the 16 chimeric viruses encompassing the SHIVenv_B pool used to infect macaques. Again the fitness difference in PBMCs did not provide evidence of significantly different Env_B3 phenotype compared to the other AHI Env_B viruses in the pool that did not establish macaque infection.

### Comparison of glycosylation and V3 net charge in the AHI Env clones

**Fig. 10:**
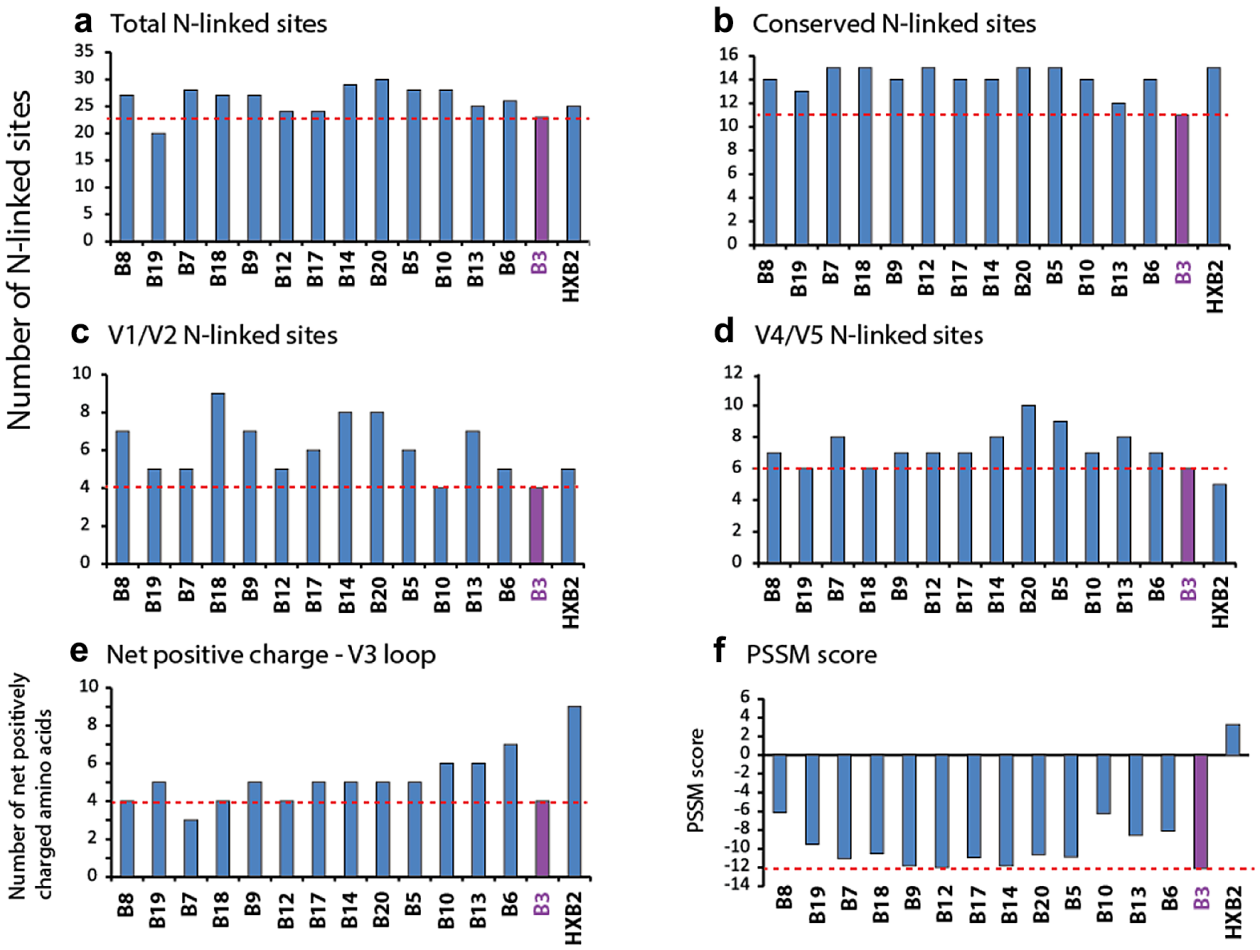
Characterization of *env*B3 and other *env* clones in SHIVenv_B pool. Comparison of putative N-linked glycosylation sites in the HIVenv_B sequences (**a**), in conserved region (**b**), in V1/V2 region (**c**), and in V4/V5 region (**d**). Net positive charge (**e**) and PSSM score (**f**) were also calculated for the HIVenv_B sequences

Previous studies have reported that transmitted HIV-1 clones in new recipients have fewer N-linked glycosylation sites in the HIV-1 Env glycoprotein. Two inter-related hypotheses have emerged: (1) a more compact Env with reduced glycosylation may have higher replicative capacity and (2) altered and increased glycosylation on HIV is necessary for escape from humoral response. In the absence of humoral response during initial acute/early infection, a more fit, less glycosylated form of HIV-1 may be transmitted. However, we have not observed a correlation between the number and position of N-linked glycosylation sites in Env with the replicative fitness of the chimeric HIV-1env viruses in primary human T cells, macrophages, or dendritic cells [37]. We compared the putative N linked glycosylation sites in our AHI Env glycoproteins and in the consensus subtype sequences. Aside from Env_B19, Env_B3 has the fewest putative (Fig. 10a) and conserved (b) N linked glycosylation sites. The reduction in N linked sites in Env_B3 was observed the V1/V2 and V4/V5 variable loops (Fig. 10c, d).

The net charge of the V3 loop and Position-Specific Scoring Matrix (PSSM) is scaling system to predict relative co-receptor usage, i.e. a low net charge and more negative PSSM score relates to CCR5 usage for HIV-1 entry into host cells. The Env_B3 has one of lowest net positive charges (Fig. 10e) and lowest negative PSSM scores (Fig. 10f) of all the other CCR5-using AHI Env’s.

## Discussion

Simian immunodeficiency viruses (SIV) or chimeras encoding the HIV-1 envelope (SHIVenv) are the most widely used in animal models of human HIV-1 infection [38–41] but both have their shortcomings for HIV-1 prevention, vaccine, and pathogenesis studies. SIVmac was initially isolated from a macaque monkey in captivity who developed an AIDS-like disease [38, 42] which prompted isolation of SIVmac_239_, a clone that causes disease progression in these monkeys [43, 44]. Subsequently, species-specific SIVs were isolated from various monkeys such as sooty mangabeys (SIVsmm) [45], African green monkeys (SIVagm) [46, 47] and mandrills (SIVmnd) [48]. These SIVs establish asymptomatic, chronic infections with slow or no progression to disease in their natural hosts. It has been suggested that SIVmac emerged via a cross-species infection of the rhesus macaque with SIVsmm naturally found in sooty mangabeys [45, 49]. SIVmac is similar to HIV-1 in genomic organization and somewhat similar in pathogenicity, e.g. depletion of CD4+ T cells in the gut early in disease [50]. Both viruses can use CCR5 as a coreceptor and target CD4+ cells such as T-lymphocytes and macrophages, resulting in the complete loss of these cells but the disease course in macaques infected with SIVmac is short relative to that of HIV-1 infections (1–3 years versus 6–10 years). Infection of macaque monkeys with SIVmac is widely used as a model for HIV/AIDS to study disease progression and virus transmission but has significant limitation for prevention and vaccine studies.

With the results of the RV144 Thailand trial [51–53] and the identification of broadly neutralizing antibodies, there is a renewed interest in humoral-based HIV vaccines as well as treatment with anti-HIV antibodies for both prevention and therapeutic use. Human trials with new vaccines or prevention strategies are typically preceded by safety and efficacy tests in macaque models. However, lack of diverse SHIVenv strains as challenge virus in macaques limits these vaccine and microbicide tests. Several vaccines tested with monovalent challenge SHIV did show complete efficacy in macaques but were less than optimal in human prevention trials [54].

In this study, we generated a pool of 16 SHIVenv’s derived from the HIV-1 *env* genes of acute HIV-1 infections. The production of these SHIVenv’s was not a simple task and first required our development of an SIV and SHIV cloning system similar to the yeast-based cloning system for HIV-1 [20, 55, 56]. Del Prete et al. [16] used conventional cloning strategy to generate a cocktail of multiple of SHIVenv viruses. This method requires unique restriction enzyme sites which is usually difficult due to the extreme diversity of HIV sequences. In order to circumvent this difficulty, we utilized a yeast based recombination technology which only relies on sequence similarity and can insert a DNA fragment in one step within yeast without the need of restriction enzyme digestion, in vitro ligation and transformations. Through yeast recombination and gap repair, we shuttled over 70 versions of HIV-1 *env* and *gp120* coding regions from 20 AHI’s into our SHIV_KB9_ vector and tested for virus production, entry efficiency, and virus propagation. Replacement of the HIV-1 KB9 with the entire *env* coding region in SHIVenv resulted in very low levels of virus production. The *env* gene carries the second exon of *tat/rev* and generates a chimera of KB9/AHI Rev and Tat proteins which appears incompatible for efficient transcription and mRNA transport. To avoid the split in *rev/tat* exons, we introduced the 20 AHI *env* genes as cassettes that included the first exon of *tat/rev* into SIVmac239 but again virus production and Env expression was too low to warrant further study. Previous studies have shown that the “KB9 Env” in the SIVmac239 has adapted to replication in macaques due in part to mutations in the extracellular domain of gp41 [57], i.e. a region that would be replaced by rev/tat-env cassette. In the end, cloning of the gp120 and partial gp41 (upstream to the second exon of rev/tat) from these AHI’s into SHIV_KB9_ preserved the KB9 Rev/Tat proteins and gp41 extracellular domain, and resulted in high level virus production from proviral transfections. These viruses (termed SHIVenv_B1 to _B20 for simplicity) had the correct stoichiometry of SIV proteins and fully functional HIV-1 glycoproteins but were unable to maintain long-term replication in various cell lines and primary, activated PBMCs of human or macaque origin.

SHIVenv_B viruses could support transient infection of different cell lines and primary macaque PBMCs but could not be propagated. Several groups have shown that propagation of SHIVenv in these primary cells or cell lines in not indicative of subsequent infection or pathogenesis in macaques [13, 30, 31]. In fact, the original SHIVenv_89.6 clone did not replicate in culture but could infect macaques and is the progenitor of the CXCR4-using SHIVenv_KB9 virus. These observations were the primary reason to proceed with a pooled SHIVenv_B exposure in macaques. Based on Env function, viral protein content, and transient replication, we selected 16 of 20 SHIVenv_B viruses for this pool. Infection was observed in one of two macaques but only one of 16 SHIV viruses (i.e. SHIVenv_B3) successfully established infection. It is important to stress that the goal of this study was to establish prolonged macaque infection with SHIVenv derived from AHI and then passage through new macaques to enhance pathogenicity. We sequenced the entire SHIVenv_B genomes from each of the 16 clones and did not observe any mutations in the SHIV_KB9_ backbone prior to infection. Furthermore, the SHIVenv_B3 during infection of m328-08 only had stochastic non-synonymous substitutions without dominant amino acid substitutions in the coding sequence across the entire proteome (including the env_B3). As reported in [19], SHIVenv_B3 did establish a long term pathogenic infection when passaged from m328-08 to m165-05 but this enhanced virulence was associated with discrete amino acid substitution in Env_B3 appearing only in the m165-05 animal. Finally, we confirmed that SHIVenv_B3 alone could establish infection in three other macaques and did not require the presence of 15 other SHIVenv_B viruses in the pool.

Infection with only one of 16 SHIVenv_B clones could be due to a stochastic/random event or may be related to distinct properties of SHIVenv_B3 compared to the other AHI *env* clones within the isogenic SHIV_KB9_ backbone. Since the SHIVenv_B viruses could not replicate long term in culture, we constructed the equivalent the HIVenv_B chimeric viruses derived from the same 16 AHI *env* genes but within an NL4-3 backbone. Despite the different SIV and HIV-1 backbones, we observed a direct correlation between the relative levels of cell fusion mediated 16 Env_B glycoproteins in the HIV-1 versus SIV backbone. A previous study involving macaque infections with a pool of SHIVenv viruses indicated that the SHIVenv with highest replication efficiency established infection in macaques [16]. In both SHIVenv monoinfections the macaque PBMCs and in competitive fitness assays using human PBMCs and HIV-1env counterparts, the Env_B3 did not show enhanced replicative efficiency. The most fit Env_B16 was not detected in the infected macaque and neither was 12 other AHI Env’s that had higher replicative fitness and/or entry efficiency. The Env_B3 mediated fourth (of 16) highest level of host cell entry as determined by Veritrop. Cell fusion was with these 16 AHI Env_B glycoproteins as compared to cell fusion mediated with another set of 26 HIVenv chimeric viruses derived from AHI cohort at Aaron Diamond/New York University [25], i.e. a pool of AHI that encompassed those SHIVenv employed by Del Prete et al. [16]. When analyzing free virus using the Affinofile system [24, 25], we found that the HIVenv chimeric viruses from CHAVI AHI and from the NYU AHI showed a wide range of affinity, avidity, and usage of CD4 and CCR5 for host cell entry. HIV-1 with Env_B3 was more efficient than the majority at host cell entry at high CCR5 across of range of CD4 levels on the cell surface but was only average when scavenging for low levels of CCR5. When comparing the kinetics of host cell entry, the HIVenv_B3 was slightly faster at these early steps of replication, ranking 3rd of the 16 HIVenv_B3 strains. However, HIVenv_B3 had lower than average replicative fitness when compared to the other 15 HIVenv_B viruses, all of which were competed against three control strains in human PBMCs. Our previous study with these HIVenv chimeric viruses also showed that HIVenv_B3 had no replicative advantage in primary macrophages, dendritic-T cell co-cultures, or infection of vaginal tissue [37]. This array of phenotypic assays suggests that HIVenv_B3 is only average in terms of replicative fitness and host cell entry.

According to a recent study [17], residue at position 375 is important in determining HIV-1 Env binding affinity to macaque CD4, i.e. S375 Env had minimum binding affinity, while mutation of S375M, Y, H, W, and F could significantly enhance the RhCD4 binding affinity and subsequent viral replication in macaque primary CD4 T cells. In our study, the 11 of 16 SHIV strains in the inoculating pool had an S375 which was associated with lower RhCD4 binding affinity. A375 and I375 were found in SHIVenv_B5 and _B12 While four strains had T375 (B16, B17 and B18). None of these residues at 375 were associated with high RhCD4 affinity. An earlier study by Boyd et al. [18] reported that independent introduction of the A204E and G312V mutations yielded functional HIV-1 Env glycoproteins on SIV capable of mediating cell infection via huCD4 and RhCD4 receptors. None of our 16 strains had either 204E or 312V. In our study, we did not deplete macaque CD8+ T cells or alter the acute/early HIV-1 *env* genes cloned into the SHIVenv_KB9 backbone. Our intention was to select for the HIV-1 *env* gene (derived from acute infections) with the highest transmission efficiency in rhesus macaques and providing the best native HIV-1 *env* for subsequent SHIVenv studies on macaque infection and pathogenesis. It is also possible that in vitro adaptation step might help to find an effective SHIVenv strain.

Asmal et al. [58] previously showed that there is a strong association between a positively charged amino acid like histidine at position 12 in transmitted/founder viruses with more efficient trafficking of the nascent envelope polypeptide to the endoplasmic reticulum and higher steady-state glycoprotein expression compared to viruses that have a non-basic position 12 residue, a substitution that was enriched among viruses sampled from chronically infected individuals. In the present study, the majority of 16 AHI viruses derived Envs including B3 contain histidine or other positively charged amino acids (e.g. Arginine) at position 12, and only B8 and B18 have non-basic amino acids at the same position. However, when comparing the Env sequences from these 16 AHIs, we did discover that the Env_B3 had the fewest conserved N-linked glycosylation sites in V1/V2 and V4/V5 loops and in *env* gene overall. The Env_B3 also had the least positively charged V3 loop (aside from B7) and the lowest PSSM score, two measures predictive of relative CCR5 versus CXCR4 usage. Increased high-mannose N-linked glycosylation accounts for 50% of the Env glycoprotein mass, can reduce the viral antigenicity and protect functional regions of Env from host antibodies [59–64]. Previous studies have shown that HIV-1 derived from acute/early infection typically have fewer N-linked glycosylation sites [65, 66]. We propose that based on the further reduction of conserved N-linked sites, the Env_B3 may have 30% less high mannose glycans than even other AHI Env’s and up to 40% less than the average Env glycoprotein found during chronic disease. The role of Env glycosylation on HIV-1 transmission still remains unclear. Early reports suggested that high transmission efficiency of HIV-1 with reduced glycosylation was related to a compact Env glycoprotein where interactions with CD4/CCR5 and subsequent confirmation changes was not impeded by the bulk high mannose glycans. Since anti-Env antibodies do not appear for 2–3 weeks post infection, there would be no selection to maintain the highly glycosylated Env on the transmitted HIV-1 strains. However, we have no evidence to suggest an increased receptor binding, host cell entry, or replicative fitness of HIV-1 with fewer N-linked sites in Env. In contrast, the Env glycoproteins from chronic disease and with more N-linked sites typically have higher replicative fitness [25]. Some SIV transmission studies showed that activated CD4 cells but not DCs are the initial targets of infection [67–69]. It has been reported that reduced glycosylation of Env may enhance binding to α4β7 integrin found on gut CD4+ T cells, which may then be associated with rapid depletion of this Th17 T cells in the gut during early HIV-1 infection [70]. On the other hand, glycans have been implicated in viral transmission through interaction with lectins, in particular the C-type lectin DC-SIGN, which is found on dendritic cells (DCs) and specific macrophages, and is thought to aid the transport of virus to anatomical sites rich in CD4+ T cells, such as lymph nodes [71, 72]. The observation of transmitted HIV with fewer N-linked sites has always run counter to the role of DC sign in transmission. In addition, binding of HIV-1 to C-type lectin Langerin, found on the surface of Langerhans’ cells is now associated with endocytosis and degradation of HIV-1 [71]. Langerhans’ cells are the primary DCs found in mucosal tissue. Regardless of these mechanisms, it is important to stress that selective transmission of the SHIVenv_B3, with the fewest N-linked sites, followed intravenous injection and not through exposure through a mucosal route. Thus, we can only speculate the role of various mechanisms on the selection of SHIVenv with the least glycosylated Env.

## Conclusions

We utilized the *env* gene from 20 acute HIV-1 infections to construct over 70 SHIVenv viruses using our yeast based cloning system. Based on reiterative cloning of various cassettes, we discovered that the selective introduction of a gp120 coding region into a SHIVenv_KB9 construct resulted in virus production with a functional Env. Despite the inability to propagate in primary cells and cell lines, a pool of 16 SHIVenv viruses could establish infection but only one virus, SHIVenv_B3 was isolated in the macaque and then shown to repeatedly infected macaques. This SHIVenv_B3 virus did not show any distinct phenotypic property from the other 15 SHIVenv viruses but did have the fewest N-linked glycosylation sites. As described in the companion article, the SHIVenv_B3 from the m328-08 animal was passaged to the m165-05 where it established a prolonged and pathogenic infection associated with distinct amino acid changes in the Env glycoprotein. We propose that the *env* genes from AHI have already undergone a selection for efficient transmission in human hosts. The SHIVenv_B3 may be the best of these AHI Envs for transmission in macaques and may provide the virus template to achieve both efficient infection and evolution to pathogenic CCR5-using SHIV.

## Additional files

**Additional file 1: Figure S1.** Schematic of production of pREC_nfl_SHIV_KB9_Δenv/URA3. Stepwise cloning scheme to introduce the near full length SHIV_KB9_ genome into the pREC_nfl_HIV-1_NL4-3_ shuttle vector. Step 1 involves amplification of the *URA3* gene from pRS316 for insertion into a digested pREC_nfl_HIV-1_NL4-3_ to generate pREC_nfl_HIV-1Δ5′HIV/URA3 via yeast homologous recombination. The latter vector was then digested with *Sac*II. A PCR product containing the first half of SHIV_KB9_ from the primer binding site to the middle of the *pol* gene was inserted (step 2). *URA3* fragment was inserted into pREC_nfl_5′SHIV_KB9_/3′HIV-1_NL4-3_ digested with *Nhe*I to generate pREC_nfl_5′SHIV_KB9__Δ3′HIV-1/URA3 (step 3). The second half of SHIV_KB9_ was subsequently PCR-amplified and inserted to form pREC_nfl_SHIV_KB9_ vector (step 4). Finally, *URA3* fragment was used to replace *env* gene in the produced pREC_nfl_SHIV_KB9_ to obtain pREC_nfl_SHIV_KB9__Δenv/URA3 vector for creation of various chimeric SHIVenv viruses.

**Additional file 2: Figure S2.** Testing of virus production from SHIV_KB9_ vector system. To test the SHIV_KB9_ vector system, the pREC_nfl_SHIV_KB9_ and the complementary vector, pREC_cplt_R/U5/gag were co-transfected into 293T cells to produce SHIV_KB9_ virus in cell-free supernatant, which was then used to infect 174×CEM.CCR5 cells. SHIV_KB9_ virus production from 174×CEM.CCR5 cells is shown in (A) and is consistent with the previous reports of SHIV_KB9_ virus propagation by Reimann et al. [30]. SHIV_KB9_ virus was harvested from day 16 supernatant of the 174×CEM.CCR5 infection and used to infect U87.CD4.CCR5 cells (B).

## Abbreviations

HIV-1: human immunodeficiency virus type 1
SHIV: Simian/human immunodeficiency virus
AHI: acute HIV-1 infections
