# Supplementary figures and images for: Infection of rhesus macaques with a pool of simian immunodeficiency virus with the envelope genes from acute HIV-1 infections
Source: AIDS Res Ther. 2016 Nov 25;13:41. doi: 10.1186/s12981-016-0125-8 (PMC5124249; doi:10.1186/s12981-016-0125-8)

Supplementary figure 1.

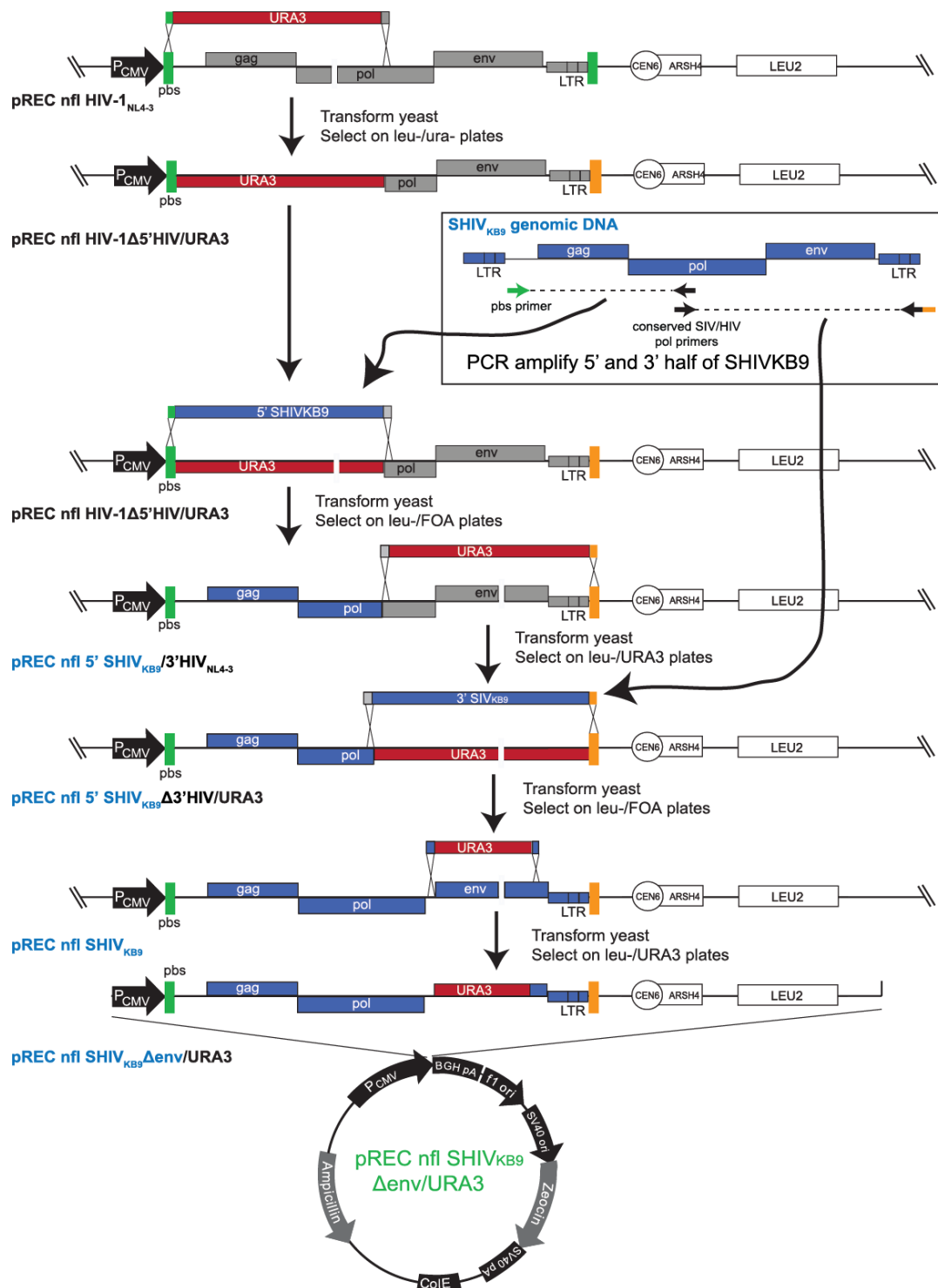

Supplement: Supplementary file 1 — Additional file 1: Figure S1. Schematic of production of pREC_nfl_SHIVKB9Δenv/URA3. Stepwise cloning scheme to introduce the near full length SHIVKB9 genome into the pREC_nfl_HIV-1NL4-3 shuttle vector. Step 1 involves amplification of the URA3 gene from pRS316 for insertion into a digested pREC_nfl_HIV-1NL4-3 to generate pREC_nfl_HIV-1Δ5′HIV/URA3 via yeast homologous recombination. The latter vector was then digested with SacII. A PCR product containing the first half of SHIVKB9 from the primer binding site to the middle of the pol gene was inserted (step 2). URA3 fragment was inserted into pREC_nfl_5′SHIVKB9/3′HIV-1NL4-3 digested with NheI to generate pREC_nfl_5′SHIVKB9_Δ3′HIV-1/URA3 (step 3). The second half of SHIVKB9 was subsequently PCR-amplified and inserted to form pREC_nfl_SHIVKB9 vector (step 4). Finally, URA3 fragment was used to replace env gene in the produced pREC_nfl_SHIVKB9 to obtain pREC_nfl_SHIVKB9_Δenv/URA3 vector for creation of various chimeric SHIVenv viruses. [file 12981_2016_125_MOESM1_ESM.pdf]

Supplementary figure 2.

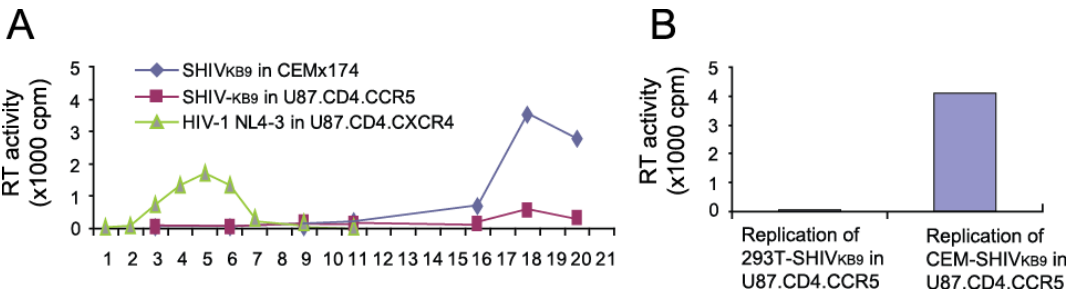

Supplement: Supplementary file 2 — Additional file 2: Figure S2. Testing of virus production from SHIVKB9 vector system. To test the SHIVKB9 vector system, the pREC_nfl_SHIVKB9 and the complementary vector, pREC_cplt_R/U5/gag were co-transfected into 293T cells to produce SHIVKB9 virus in cell-free supernatant, which was then used to infect 174×CEM.CCR5 cells. SHIVKB9 virus production from 174×CEM.CCR5 cells is shown in (A) and is consistent with the previous reports of SHIVKB9 virus propagation by Reimann et al. [30]. SHIVKB9 virus was harvested from day 16 supernatant of the 174×CEM.CCR5 infection and used to infect U87.CD4.CCR5 cells (B). [file 12981_2016_125_MOESM2_ESM.pdf]
